# Non-host Plant Resistance against *Phytophthora capsici* Is Mediated in Part by Members of the *I2 R* Gene Family in *Nicotiana* spp.

**DOI:** 10.3389/fpls.2017.00205

**Published:** 2017-02-15

**Authors:** Julio C. Vega-Arreguín, Harumi Shimada-Beltrán, Jacobo Sevillano-Serrano, Peter Moffett

**Affiliations:** ^1^Boyce Thompson Institute for Plant Research, IthacaNY, USA; ^2^Laboratorio de Ciencias Agrogenómicas, Escuela Nacional de Estudios Superiores – León, Universidad Nacional Autónoma de MexicoLeón, Mexico; ^3^Département de Biologie, Faculté des Sciences, Université de Sherbrooke, SherbrookeQC, Canada

**Keywords:** *Phytophthora capsici*, *Nicotiana*, non-host resistance, *R* gene, hypersensitive response

## Abstract

The identification of host genes associated with resistance to *Phytophthora capsici* is crucial to developing strategies of control against this oomycete pathogen. Since there are few sources of resistance to *P. capsici* in crop plants, non-host plants represent a promising source of resistance genes as well as excellent models to study *P. capsici* – plant interactions. We have previously shown that non-host resistance to *P. capsici* in *Nicotiana* spp. is mediated by the recognition of a specific *P. capsici* effector protein, PcAvr3a1 in a manner that suggests the involvement of a cognate disease resistance (R) genes. Here, we have used virus-induced gene silencing (VIGS) and transgenic tobacco plants expressing dsRNA in *Nicotiana* spp. to identify candidate *R* genes that mediate non-host resistance to *P. capsici*. Silencing of members of the *I2* multigene family in the partially resistant plant *N. edwardsonii* and in the resistant *N. tabacum* resulted in compromised resistance to *P. capsici*. VIGS of two other components required for *R* gene-mediated resistance, *EDS1* and *SGT1*, also enhanced susceptibility to *P. capsici* in *N. edwardsonii*, as well as in the susceptible plants *N. benthamiana* and *N. clevelandii*. The silencing of *I2* family members in *N. tabacum* also compromised the recognition of PcAvr3a1. These results indicate that in this case, non-host resistance is mediated by the same components normally associated with race-specific resistance.

## Introduction

*Phytophthora capsici* is a soil-borne oomycete that causes Phytophthora blight disease in many solanaceous and cucurbit plants worldwide (Hausbeck and Lamour, 2004; Tian and Babadoost, 2004; Lamour et al., 2012b). With more than 50 different plant species reported as hosts of *P. capsici*, and the increasing incidence of this disease, the development of resistance strategies is becoming an important challenge. In affected plants, the typical symptoms of the disease are wilting and a root and crown rot characterized by a dark brown stem lesion extending upward from the soil line. The infection can rapidly progress in appropriate conditions leading to the death of the plant. Despite increasing incidence of *P. capsici*, sources of genetic resistance in the most affected crop plants (pepper, eggplant, and cucurbits) are largely absent. Research on *P. capsici* has been enhanced by the sequencing of its genome (Joint Genome Institute^1^) and it is emerging as a model system for the study of host–oomycete interactions.

Plant disease resistance in which all accessions or cultivars of a plant species are resistant to all strains of a pathogen species is often referred to as non-host resistance. Non-host resistance allows most plants to resist most pathogens, although this broad classification is likely the manifestation of many different molecular mechanisms. This is in apparent contrast to race-specific, or gene-for-gene, disease resistance wherein dominant plant disease resistance (*R*) genes confer resistance to specific pathogens encoding matching Avirulence (*Avr*) genes (Dangl and Jones, 2001). *R* gene mediated resistance is an active process whereby recognition of pathogens results in the activation of multiple signaling pathways and often culminates a form of programmed cell death known as the hypersensitive response (HR) (Mur et al., 2008). Most *Avr* genes encode pathogen effector proteins and this phenomenon is now more commonly referred to as effector-triggered immunity (ETI) (Chisholm et al., 2006; Jones and Dangl, 2006). Plant *R* genes are highly polymorphic both within and between populations (Clark et al., 2007; Ding et al., 2007; Yang et al., 2008), and effective *R* genes are often introgressed into crop plants from wild relatives. *Avr* genes are also frequently polymorphic and *R* genes may confer resistance to most pathogen or all pathogen races, isolates, or strains, or they may only be effective against a small subset. As such, some *R* genes are durable over long periods of deployment, whereas others are quickly rendered ineffective due to newly emerged or introduced “resistance-breaking” strains (Harrison, 2002; McDonald and Linde, 2002; Parlevliet, 2002).

The majority of plant *R* genes encode for NB-LRR (Nucleotide Binding Site–Leucine-Rich Repeat) proteins. There are three major classes of NB-LRR proteins, distinguished by the protein domain encoded at the N terminus; either a Toll and Interleukin-1 Receptor homology domain (TIR; TIR-NB-LRRs), a loosely predicted coiled-coil domain (CC; CC-NB-LRRs) or a CC domain with homology to RPW8 (CC_R_; CC_R_-NB-LRR) (Collier et al., 2011; Shao et al., 2016). NB-LRR proteins recognize effector/Avr proteins from all types of pathogens and several *R* genes conferring resistance to members of the *Phytophthora* genus have been described including *Rb/RpiBlb*1, *R*1, *R3b*, and *R3a*, which confer resistance to *P. infestans* (Ballvora et al., 2002; Song et al., 2003; van der Vossen et al., 2003; Armstrong et al., 2005; Li et al., 2011; Oh et al., 2014), and *Rps1k, Rps*4, and *Rps*6 which confer resistance to *P. sojae* (Sandhu et al., 2004; Gao et al., 2005). The potato resistance protein R3a was shown to recognize AVR3a from *P. infestans* (Armstrong et al., 2005; Bos et al., 2006) (Engelhardt et al., 2012). Also, silencing of Avr3a compromises *P. infestans* pathogenicity, indicating an important role of Avr3a in virulence (Bos et al., 2010).

The tomato *I2* gene, a CC-NB-LRR R-gene that was shown to confer resistance to race 2 of *Fusarium oxysporum* f sp. *lycopersici* (Ori et al., 1997; Simons et al., 1998), is a homolog of the potato *R3a* gene that confers resistance to *P. infestans* (Huang et al., 2004; Armstrong et al., 2005) as well as the *L* genes of pepper and the *N’* gene of tobacco, which confer resistance to tobamoviruses (Tomita et al., 2011; Sekine et al., 2012). Genes encoding I2-like proteins appear to be monophyletic clade specific to Solanaceous species, where they have expanded significantly in all the species investigated, including tobacco (Couch et al., 2006; Seo et al., 2016). Expression of the CC domain of I2-like proteins in *Nicotiana benthamiana* leaves is sufficient to induce an HR, whereas the LRR domain is thought to confer recognition specificity (Tomita et al., 2011; Sekine et al., 2012; Chapman et al., 2014; Hamel et al., 2016).

Plant signaling components important for defense against a variety of pathogens have also been identified. EDS1 (*enhanced disease susceptibility* 1) encodes a protein with homology to lipases that is an essential component of basal resistance to invasive biotrophs and certain hemibiotrophs (Parker et al., 1996; Wiermer et al., 2005). EDS1 is also required for the activity of the TIR-NBS-LRR class of *R* genes (Aarts et al., 1998; Hu et al., 2005). The *SGT1* (suppressor of the G2 allele of *skp1*) gene is essential for the function of most *R* genes (Muskett and Parker, 2003; Azevedo et al., 2006). The SGT1 protein appears to play a role in NB-LRR protein folding and accumulation (Holt et al., 2005; Azevedo et al., 2006) and elimination of SGT1 likely affects the activity of most or all NB-LRR proteins (Liu et al., 2002b; Peart et al., 2002b).

In this study, we used a candidate-gene approach to identify genes important for disease resistance to *P. capsici*. We have previously demonstrated that *P. capsici* Avr3a1 (PcAvr3a1) is recognized in several *Nicotiana* species and that such recognition is associated with resistance (Vega-Arreguin et al., 2014). Since PcAvr3a1 is a homolog of *P. infestans* Avr3a, which is recognized by the I2-like potato protein R3a gene, we hypothesized that a tobacco I2-like protein might confer resistance to *P. capsici* through the recognition of PcAvr3a1. Here, we have evaluated resistance to *P. capsici* in different *Nicotiana* species amenable to virus-induced gene silencing (VIGS) studies. Tobacco Rattle Virus (TRV) – based VIGS of *I2-like, EDS1*, and *SGT1* was used to investigate their possible role in non-host resistance against *P. capsici* in several *Nicotiana* species. Transgenic *N. tabacum* expressing an *I2* hairpin construct for RNAi were also generated to evaluate the role of this gene in resistance. Differences in susceptibility to *P. capsici* were compared between silenced versus non-silenced plants upon inoculation with *P. capsici* zoospores. Our results indicate a role for I2-like proteins in the recognition of PcAvr3a1 and resistance to *P. capsici* as well as a role for EDS1 and SGT1 in basal resistance in susceptible plants.

## Results

### Identification of a *P. capsici* Resistant *Nicotiana* Species Amenable to TRV-Based Virus-Induced Gene Silencing

**FIGURE 1:**
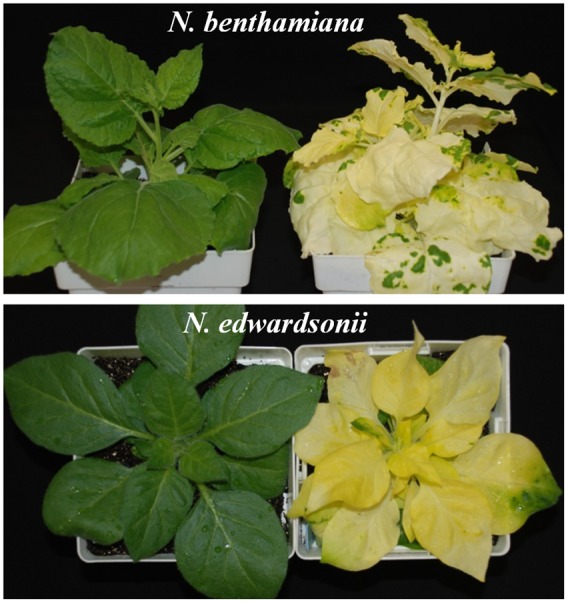
**Efficiency of TRV-based VIGS in *N. edwardsonii*.** Three-week-old *N. edwardsonii* and *N. benthamiana* plants were agro-infiltrated with *Agrobacterium* carrying a fragment of the *N. benthamiana Sulfur* (magnesium chelatase) gene cloned in TRV00 along with *Agrobacterium* carrying pBintra6 (RNA I) (right). Photographs were taken 4 weeks post-infiltration.

We have previously tested a number of *Nicotiana* species for their susceptibility or resistance to *P. capsici* based on application of zoospores to leaves (Vega-Arreguin et al., 2014). In order to use this information for reverse genetics, we tested a number of *Nicotiana* species for their amenability to TRV-based VIGS using the pTV00 vector (Ratcliff et al., 2001) carrying a fragment of the *N. benthamiana Sulfur* gene (encoding magnesium chelatase) (Kjemtrup et al., 1998; Lu et al., 2003). Supplementary Table S1 lists *Nicotiana* species amenable to TRV VIGS and **Figure 1** shows a *Sulfur*-silenced *N. edwardsonii* plant 4 weeks post-infiltration.

To follow up these observations, we tested the response of a subset of VIGS-amenable *Nicotiana* species, *N. benthamiana, N. clevelandii*, and *N. edwardsonii*, to soil inoculation of zoospores. The New York isolate B1-3.1 of *P. capsici* was used for the infection assays. Four- to five-week-old plants were challenged with a *P. capsici* zoospore suspension and monitored for disease symptoms at 10 and 14 days post-inoculation (dpi). We found that *N. benthamiana* and *N. clevelandii* were highly susceptible to infection by *P. capsici*, similar to what was previously observed with detached leaves (Vega-Arreguin et al., 2014). At 10 and 14 dpi all the *N. benthamiana* and *N. clevelandii* plants showed 80 to 100% of infection, whereas *N. edwardsonii*, a hybrid derived from a cross between *N. glutinosa* and *N. clevelandii* (Christie, 1969), showed only partial susceptibility with 14.7% of the plants showing 80–100% of infection at 14 dpi (**Table 1**). Six other isolates of *P. capsici* from New York State: 06120-1, 0664-1, 0752-2, 0759-8, A3-3.1, and 8.2-1 were also tested on *N. benthamiana* with similar results (data not shown). Infection experiments with other *Nicotiana* species have shown that *N. glutinosa*, as well as *N. tabacum*, are resistant to *P. capsici* (**Table 1** and Vega-Arreguin et al., 2014), but these species do not support TRV-based VIGS efficiently. Hence, the relatively weak susceptibility of *N. edwardsonii* to *P. capsici* makes it a good candidate for VIGS–based studies of resistance against *P. capsici*.

**Table 1:** *P. capsici* infection in *N. benthamiana, N. clevelandii, N. edwardsonii*, and *N. tabacum.*

	10 dpi				14 dpi			
	0^∗^	1	2	3	0	1	2	3
*N. benthamiana* (*N* = 30)	0^a^	0	0	100	0	0	0	100
*N. clevelandii* (*N* = 30)	0	0	0	100	0	0	0	100
*N. edwardsonii* (*N* = 61)	86.9	11.5	1.6	0	60.7	11.5	13.1	14.7
*N. tabacum* (*N* = 30)	100	0	0	0	100	0	0	0

*^∗^Scale of infection: **0** = Healthy; **1** = wilted or 1–20% of the plant infected; **2** = 21–80% of the plant infected; **3** = 81–100% of the plant infected. ^a^Numbers are the % of the total (N) plants tested.*

### Silencing of the Tobacco *I2* Family Enhances Disease Susceptibility to *P. capsici in N. edwardsonii* and *N. tabacum*

**FIGURE 2:**
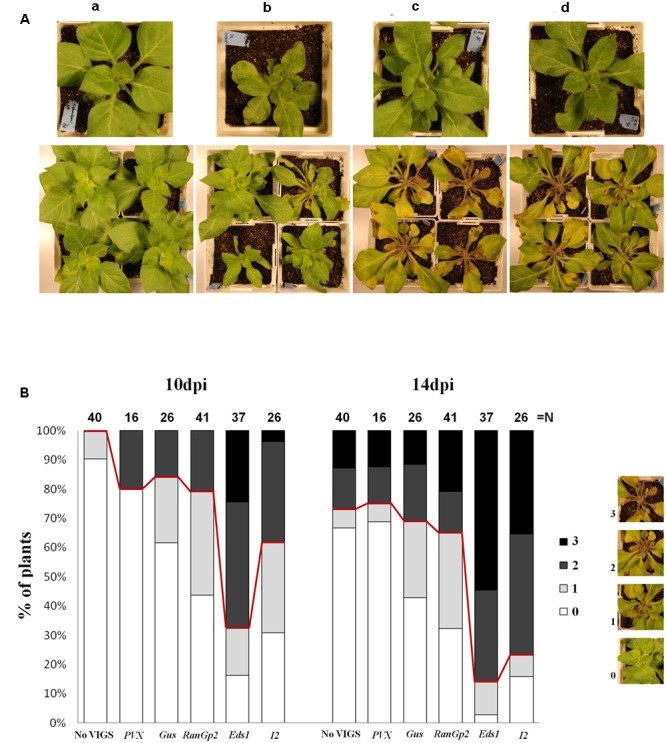
**Virus-induced gene silencing of *EDS1* and *I2* enhances susceptibility to *P. capsici* in *N. edwardsonii*.** Three-week-old plants were infiltrated with *Agrobacterium* carrying pTRV2-*EDS1* or pTRV2-*I2* along with *Agrobacterium* carrying TRV1. At 3 weeks post-infiltration plants were challenged with 5 × 10^5^ zoospores of *P. capsici* inoculated in the soil near the base of the plant. **(A)** Top panel are agro-infiltrated plants right before inoculation with *P. capsici*. Bottom panel are the plants inoculated with *P. capsici* at 10 dpi. Control non-infiltrated plants **(a)**, agro-infiltrated with pTRV2-*Gus* **(b)**, pTRV2-*EDS1* **(c)** or pTRV2-*I2* **(d)**. Experiments were repeated three times with similar results and representative pictures are presented. **(B)** Quantification of *P. capsici* infection in VIGSed *N. edwardsonii* plants. Controls were non-infiltrated plants or infiltrated with pTRV2-*Gus*, pTRV2-*RanGp2*, and PVX-based vector pGR107. Symptoms were monitored at 10 and 14 dpi according to the scale of infection as follows: **0** = Healthy; **1** = 1–20% of the plant affected, Slightly Diseased; **2** = 21–80% of the plant affected, Heavily Diseased; **3** = 81–100% of the plant affected, Dead. N indicate the total number of plants tested from three independent experiments.

Since *P. capsici* Avr3a1 triggers an HR in tobacco and other *Nicotiana* species and such recognition correlates with resistance to *P. capsici* (Vega-Arreguin et al., 2014), and the tomato I2 protein can weakly recognize *P. infestans* Avr3a (Giannakopoulou et al., 2015), we hypothesized that a homolog of *I2* in tobacco could be mediating such resistance to *P. capsici*. To determine whether the tobacco *I2* gene family is involved in resistance to *P. capsici*, we used VIGS in *N. edwardsonii* and RNAi in stably transformed *N. tabacum* to silence most of the members of the family by targeting the highly conserved NBS region of *I2*. In a previous study, Couch et al. (2006) sampled 44 distinct *I2*-NBS sequences from tobacco. For VIGS of *I2* we amplified and cloned in sense orientation into the pTRV2 vector (Liu et al., 2002a) a 840 bp sequence corresponding to the NBS region of *I2* from tobacco that shows high similarity with those reported previously (Couch et al., 2006) (**Supplementary Figure S1**). pTRV2-*I2* was agroinfiltrated along with TRV1 in *N. edwardsonii* for VIGS of the *I2* gene family. pTRV2-*Gus* (Tameling and Baulcombe, 2007) and non-virus-infected plants served as controls. **Figure 2A** shows the *I2*-silenced *N. edwardsonii* plants before and after infection with *P. capsici.* The *I2*-silenced plants were more susceptible to *P. capsici* when compared with the control plants. At 10 dpi 30.8% of the plants did not present visible symptoms, compared to 61.5% in the control with pTRV2-*Gus* and 90% in the non-virus-infected plants (**Figure 2B**). At 14 dpi only 15.8% of the pTRV2-*I2* infected plants were healthy and the controls with or without pTRV2-*Gus* presented 42.3 and 65% of healthy plants, respectively. Similar results were obtained when another sequence (also cloned in sense orientation into pTRV2) corresponding to the conserved NBS-*I2* region chosen from the population of amplified PCR products was used for VIGS (data not shown), indicating that the silencing and phenotype derived thereof can occur with more than one NBS-*I2* sequence.

It is noteworthy that TRV infection with pTRV2 carrying a fragment of *Gus* or *RanGap2* (Tameling and Baulcombe, 2007) has some effect on *P. capsici* infection when compared with the “non-VIGS” (non-virus-infected) plants (**Figures 2A,B**) indicating that TRV infection itself appears to moderately enhance susceptibility to *P. capsici*. To test whether infection with a different virus has a similar response to the inoculation with *P. capsici*, plants were infected with *Potato virus X* (PVX) 3 weeks before inoculation with *P. capsici*. Contrary to that observed with TRV, plants infected with PVX were as resistant to *P. capsici* as the “non-VIGS” plants (**Figure 2B**). Thus, assuming that the silencing is specific, the enhanced susceptibility to *P. capsici* in *N. edwardsonii* is mainly due to the silencing of *I2*.

**FIGURE 3:**
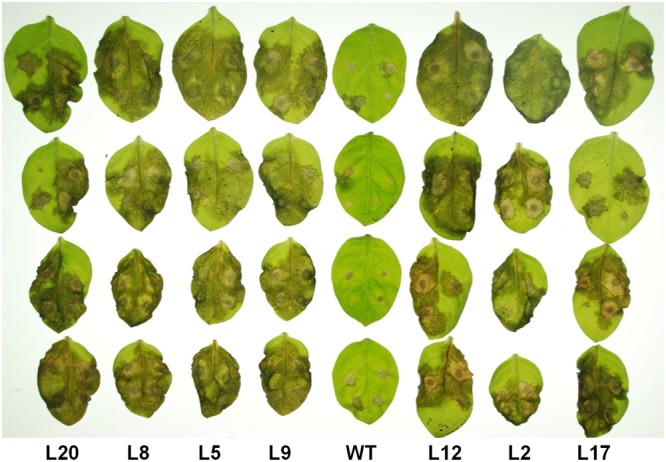
**Resistance to *P. capsici* is compromised in transgenic tobacco leaves expressing a RNAi construct to silence *I2* genes.** Infection assay with *P. capsici* in transgenic tobacco lines expressing a hairpin of *I2* was carried out in detached leaves of 4-week-old plants. Representative leaves from wild-type (WT) and different transgenic lines (L20, L8, L5, L9, L12, L2, and L17) inoculated with *P. capsici* zoospores are shown. Photographs were taken at 3 dpi.

To study the role of I2 against *P. capsici* in the resistant *N. tabacum*, we used the same NBS-*I2* region used for VIGS to generate stable transformed plants with a hairpin construct to silence the *I2* gene family by RNAi. The NBS-*I2* region was amplified and cloned into the pHellsgate vector (Wesley et al., 2001) in sense and antisense orientations. Transformation of *N. tabacum* plants with this construct generated 20 independent transformed lines. Transformed plants and detached leaves were inoculated either with a zoospore suspension and/or mycelium plugs of *P. capsici*, and infection development was monitored 2 to 6 days after inoculation. **Figure 3** shows the detached leaves of wild-type (WT) and transformed plants inoculated with *P. capsici*. Detached leaves of *N. tabacum* transformed with an *I2* hairpin construct were more susceptible to *P. capsici* when compared to WT leaves (**Figure 3**). Similar results were obtained when detached leaves were inoculated with agar plugs containing mycelium of *P. capsici* (**Supplementary Figure S3**). Also, *I2* hairpin transgenic *N. tabacum* plants inoculated in the soil near the stem with *P. capsici* zoospores were more susceptible than the WT plants (**Supplementary Figure S4**). From these data, we conclude that a member of the multigene *I2* family is important for resistance to *P. capsici* in *N. edwardsonii* and *N. tabacum.*

### Silencing of *EDS1* Enhances Disease Susceptibility to *P. capsici*

EDS1 has been shown to play a crucial role in basal defenses of plants and R-gene mediated resistance against different pathogens (Parker et al., 1996; Peart et al., 2002a; Bhattacharjee et al., 2011; Rietz et al., 2011; Moreau et al., 2012; Gao et al., 2014). To determine whether EDS1 is involved in resistance to *P. capsici* we silenced *EDS1* in *N. edwardsonii* using VIGS. A pTRV2 vector containing a heterologous fragment of *EDS1* gene from tobacco (Liu et al., 2002a) was inoculated by agroinfiltration along with TRV1 in 3-week-old *N. edwardsonii* plants. pTRV2-*Gus* was used as a control. Three to four weeks after infiltration with pTRV2-*EDS1* the plants were challenged with *P. capsici* zoospores and the infection was monitored at 10 and 14 dpi. **Figures 2A,B** show the results of the *EDS1* silencing experiment. At 10 and 14 dpi *EDS1*-silenced plants showed enhanced disease susceptibility to *P. capsici*. The percentage of plants with no visible symptoms at 10 dpi decreased to 16.2% in pTRV2-*EDS1* infiltrated plants, compared to 61.5 and 90% of healthy plants in the controls with or without pTRV2, respectively. At 14 dpi the healthy *EDS1*-silenced plants represented only 2.7% of the total plants tested, compared to 42.3 and 65% in the controls. Thus, silencing of *EDS1* in *N. edwardsonii* clearly enhanced disease susceptibility to *P. capsici*.

## Silencing of *Sgt1* Enhances Susceptibility to *P. capsici*

**FIGURE 4:**
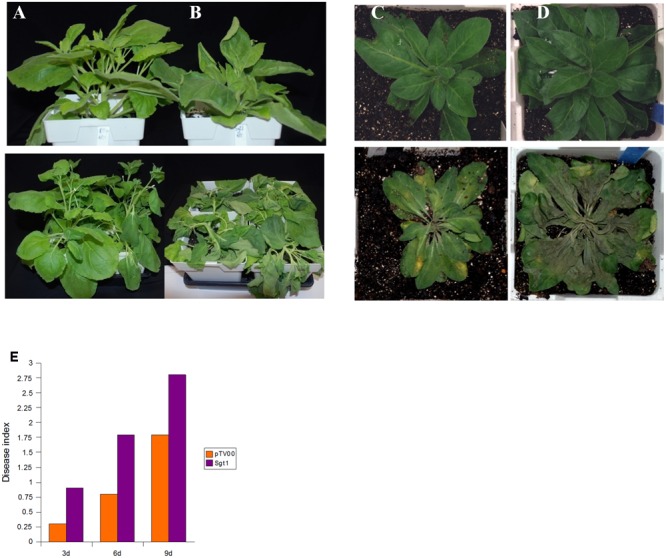
**Virus-induced gene silencing of *SGT1* enhances susceptibility to *P. capsici* in *N. benthamiana* and *N. clevelandii.*** Three-week-old *N. benthamiana* **(A,B)** and *N. clevelandii* **(C,D)** plants were subjected to TRV VIGS using TV-*SGT1* **(B,D)** or pTV00 **(A,C)**. At 3 weeks post-infiltration plants were challenged with 2 × 10^5^ zoospores of *P. capsici* inoculated in the soil near the base of the plant. Symptoms were monitored at 3, 6, and 9 dpi. Top panels are VIGSed plants right before inoculation with *P. capsici*. Bottom panels are the plants inoculated with *P. capsici* at 3 dpi. **(E)** Disease index (DI = [Σ(number of plants × category value)/total number of plants]) of the *SGT1*-silenced *P. capsici*-inoculated *N. benthamiana* plants at 3, 6, and 9 dpi. A representative experiment is presented from three independent replicates.

SGT1 (suppressor of the G2 allele of *skp1*) is required for resistance conferred by a number of R-genes (Peart et al., 2002b; Tor et al., 2002; Oh et al., 2014), and it was recently shown its role in pepper immunity against *P. capsici* upon interaction with SRC2-1 (Liu et al., 2016). We silenced this gene in *N. benthamiana* and *N. clevelandii* using VIGS. Silencing of *SGT1* in *N. edwardsonii*, but not *N. benthamiana* and *N. clevelandii*, resulted in lethality (**Supplementary Figure S2**), indicating that SGT1 is not dispensable in this species, similar to VIGS results in tomato and double knock-out mutants of *Arabidopsis SGT1a* and *SGT1b* (Muskett and Parker, 2003; Bhattarai et al., 2007). Three weeks after the initiation of VIGS, plants were inoculated in the soil near the base of the plant with 2 × 10^5^ zoospores of *P. capsici* B1-3.1. Silencing of *SGT1* in *N. benthamiana* resulted in plants more susceptible to *P. capsici* than the vector-silenced plants as evidenced by a wilting phenotype caused by *P. capsici* observed at 2 dpi in *SGT1*-silenced plants as opposed to 4 dpi in control plants (**Figures 4A,B,E**). Similar results were obtained with *N. clevelandii* where infection progressed faster in *SGT1*-silenced plants when compared to control plants (**Figures 4C,D**). These results indicate that SGT1 is important not only for *R* gene mediated resistance but also for basal resistance responses to *P. capsici*.

### *P. capsici* Avr3a1-Triggered HR Is Compromised upon Silencing of *I2* in *N. tabacum* RNAi Transgenic Plants

**FIGURE 5:**
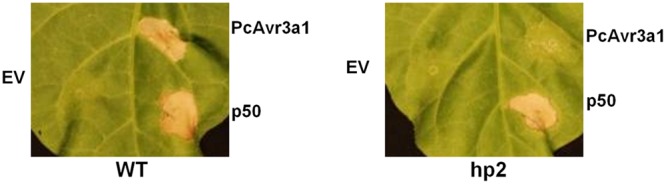
***PcAvr3a1*-triggered HR in *N. tabacum* is compromised in transgenic *I2*-silenced plants.** Leaves from 5- to 6-week-old *N. tabacum* (cv. Samsun NN) plants were infiltrated with *Agrobacterium* carrying either empty pBin61 (EV), or constructs expressing PcAvr3a1 or the TMV p50 protein, as indicated. Photographs of representative leaves of WT **(Left)** and *I2*-hairpin transgenic **(Right)** plants were taken at 5 dpi.

Since PcAvr3a1 triggers an HR in *N. tabacum* (**Figure 5**) and *N. edwardsonii* (**Supplementary Figure S5**) (Vega-Arreguin et al., 2014) we tested whether this PcAvr3a1-triggered HR is compromised in *I2*-silenced plants, either in *N. tabacum* stably transformed plants or in *N. edwardsonii* VIGS plants. *Agrobacterium* carrying PcAvr3a1 was infiltrated in leaves of *I2*-silenced plants as well as in the control plants. HR was monitored at 5–8 dpi. **Figure 5** shows the lack of PcAvr3a1 recognition in transgenic *N. tabacum* leaves agroinfiltrated with PcAvr3a1, whereas in *N. tabacum* WT leaves agroinfiltration of PcAvr3a1 triggered a HR in the infiltration site. Agroinfiltration of Tobacco Mosaic Virus (TMV) p50 served as positive control since these tobacco plants contain the N resistant gene. In all cases, p50 triggered a HR in the infiltration site, indicating that the absence of HR upon agroinfiltration of PcAvr3a1 may be specifically a consequence of the silencing of the *I2* gene family and the lack of recognition of one of its members to PcAvr3a1.

In *N. edwardsonii* VIGS plants, *Agrobacterium* carrying PcAvr3a1 was infiltrated in leaves of *EDS1*- and *I2*-silenced plants as well as in the VIGS control plants. HR was monitored at 5 dpi. **Supplementary Figure S5** shows that the percentage of the infiltrated sites showing HR is not affected by the silencing of *EDS1* and *I2* as compared with the controls, suggesting that PcAvr3a1 is being recognized by the remaining product of the silenced gene in *N. edwardsonii*, since VIGS usually reduces the level of the target transcript without completely knocking down the expression (Senthil-Kumar and Mysore, 2011). PcAvr3a does not trigger a visible HR upon agroinfiltration in *N. benthamiana* (**Supplementary Figure S5**), and silencing of *SGT1* in *N. edwardsonii* resulted in lethality (**Supplementary Figure S2**), thus the effect of silencing *SGT1* in PcAvr3a-triggered-HR was not assessed in these species. In *N. clevelandii* (which responds to PcAvr3a1 with a very weak necrosis, Vega-Arreguin et al., 2014), *SGT1*-silenced plants showed complete abolition of that PcAvr3a1-triggered necrosis (not shown), indicating that SGT1 is important for mediating recognition of PcAvr3a with its cognate *R* gene.

## Discussion

Despite the economic importance of *P. capsici*, little is known about the genes involved in compatible or incompatible interactions with plants. Although some resistance genes have been described against *P. infestans* (for instance: *R1, RB, R3a, Rpi-blb*) (Ballvora et al., 2002; Song et al., 2003; van der Vossen et al., 2003; Armstrong et al., 2005; Oh et al., 2014) and *P. sojae* (*Rps1k, Rp4, Rp6*) (Sandhu et al., 2004; Gao et al., 2005), no cloned resistance genes against *P. capsici* have been reported. Non-host resistance to *P. capsici* in several *Nicotiana* species appears to be an active resistance mechanism as opposed to passive (true incompatibility) or PTI-mediated defense as supported by our previous findings on the correlation between resistance in several *Nicotiana* species with PcAvr3a1-triggered HR (Vega-Arreguin et al., 2014). As a first step toward understanding this resistance mechanism we have used VIGS and stable transformation with an RNAi construct in *Nicotiana* spp. to study the effect of silencing a multigene NB-LRR R family and two components (EDS1 and SGT1) required for disease resistance in plants. Our results demonstrate the viability of using silencing approaches to study the basis of non-host resistance to *P. capsici* in resistant plants. In addition, since silencing of *EDS1* and *SGT1* leads to even greater susceptibility, our approach can also be used to study the contribution of various components that participate in the basal level of resistance seen in susceptible plants. In principle then, this experimental approach should also be applicable to studying host factors involved in susceptibility and is complementary to the use of HIGS (Host-Induced Gene Silencing) in *Nicotiana* to study the role of *P. capsici* genes in infection (Vega-Arreguin et al., 2014).

The experimental system *Nicotiana – P. capsici* has become of particular interest as a model for *R* gene and non-host resistance. Some advantages of this system are: (1) complete genome sequences are available for *N. tabacum* (Sierro et al., 2014), *N. benthamiana* (Bombarely et al., 2012) and *P. capsici* (Lamour et al., 2012a); (2) many *Nicotiana* species are particularly amenable to VIGS (Supplementary Table S1); (3) agro-infiltration of these plants is easy; and (4) most genes in *Nicotiana* are probably close enough in sequence that a gene sequence from one species can be used to silence in another without having to find the gene in the uncharacterized plants. In addition, VIGS can overcome gene function redundancy in multigene families by targeting a conserved region of the gene. Thus, most or all the members of the family will be silenced. Furthermore, TRV-based VIGS (Ratcliff et al., 2001; Liu et al., 2002a) has proven efficient in the identification of components involved in resistance signaling (Liu et al., 2002a, 2016; Peart et al., 2002a,b; Brigneti et al., 2004; Collier et al., 2011; Oh et al., 2014) and, as we have shown here, it can be an efficient tool to identify components of resistance signaling against soil-borne pathogens in *Nicotiana* species. Also, another relevant advantage of the *Nicotiana* – *P. capsici* system is the fact that different degrees of resistance to *P. capsici* can be observed in several *Nicotiana* species, for instance, *N. benthamiana* is susceptible, *N. edwardsonii* is partially susceptible and *N. tabacum* is resistant (**Table 1**). There are also cases of non-host resistance in *Nicotiana* species against *P. capsici* that might be different from what we have reported here, for example, *N. plumbaginifolia* is resistant to *P. capsici* but there is not a visible HR either with the pathogen or with PcAvr3a1 (Vega-Arreguin et al., 2014).

In this report, we have shown that silencing of the *I2* family resulted in enhanced susceptibility to *P. capsici* in *N. edwardsonii* and *N. tabacum*. Although further work will be required to identify the precise family member(s) that recognize PcAvr3a1, this represents the first NB-LRR *R* gene family found to be involved in resistance to *P. capsici*. Silencing of *I2* compromised the PcAvr3a1-triggered HR in *N. tabacum* stably transformed with an RNAi construct (**Figure 5**). Although the HR was not compromised in *N. edwardsonii* VIGSed plants, VIGS of I2 nonetheless compromised resistance suggesting that the residual I2 product could be recognizing PcAvr3a1, which in this assay is likely much more abundant than when delivered from the pathogen. The compromise of resistance/Avr3a1 recognition by silencing the *I2* family in three *Nicotiana* species, as well as the common occurrence of the ability to recognize Avr3a1 (Vega-Arreguin et al., 2014) suggests that this recognition is mediated by a highly conserved I2 protein homolog that has been maintained in the *Nicotiana* genus. Cloning and characterization of these *I2*-like genes from resistant *Nicotiana* species will provide insights into non-host resistance to *P. capsici*, as well as opening up the possibility of transferring this “non-host” *R* gene to susceptible Solanaceous crops.

EDS1 plays a role as a positive regulator of basal resistance but also in *R* gene mediated resistance. EDS1 is required for most cases of TIR-NB-LRR mediated resistance, although a few examples of EDS1 being required for CC-NB-LRR mediated resistance have been described. For instance, HRT-mediated resistance against Turnip Crinkle Virus (Chandra-Shekara et al., 2004). EDS1 is also required for the function of RPW8, a non-NB-LRR protein, in resistance against powdery mildew (Xiao et al., 2005). Here, we noted that EDS1 silenced *N. edwardsonii* are more susceptible than the I2 silenced plants. At this point, we cannot unequivocally conclude that EDS1 is specifically for I2 mediated resistance against P. capsici, although this possibility also cannot be ruled out. Silencing of EDS1 may render the plant more susceptible overall to the oomycete, thus reducing the effectiveness of I2-like protein function. Further experiments would be required to test these possibilities.

A hemi-biotrophic lifestyle has been suggested to occur in several *Phytophthora* species and the transition from biotrophy to necrotrophy has been recently studied for *P. capsici* (Jupe et al., 2013) and *P. infestans* (Zuluaga et al., 2016) using transcriptome sequencing during the compatible interaction with tomato plants. In both cases, transcriptional changes associated with biotrophy and the subsequent switch to necrotrophy were described (Jupe et al., 2013; Zuluaga et al., 2016). Here, we found that EDS1 and SGT1, two important components of defense against biotrophs (Parker et al., 1996; Peart et al., 2002a,b; Collier et al., 2011; Liu et al., 2016) were also important for *R* gene-mediated and basal resistance against *P. capsici*. We have shown in this study that silencing of heterologous *EDS1* and *SGT1* in diverse *Nicotiana* species enhances susceptibility to *P. capsici*. This is in contrast to what has been shown for the necrotroph *Botrytis cinerea*, where *EDS1* and *SGT1* are activated upon infection of *N. benthamiana* and the plants became resistant when these genes were silenced by VIGS (El Oirdi and Bouarab, 2007). These results suggest that the initial defenses against *P. capsici* function against the biotrophic phase of *P. capsici*. Indeed, we have previously reported that at very high inoculum, *P. capsici* can overcome the presumed *R* gene-mediated, anti-biotrophic, resistance mechanisms of *N. tabacum* (Vega-Arreguin et al., 2014). These observations are pertinent to developing plants resistant to *P. capsici* as they suggest that once the pathogen switches to a necrotrophic phase, anti-biotrophic defense mechanisms will no longer be effective.

## Materials and Methods

### *P. capsici* Isolates and Zoospore Production

*P. capsici* isolates B1-3.1, 06120-1, 0664-1, A3.1-1, 0752-2, 0758-1, 8.2-1 (kindly provided by Chris Smart, Geneva, NY, USA) were grown in 20% V8 vegetable juice agar (Campbells) complemented with 1gr CaCO3/L for 3–5 days in the dark at 26°C. Sporulation was induced by growing three additional days under continuous fluorescent light. Release of zoospores was induced by flooding the cultures with sterile deionized water and incubated at 6°C for 30–60 min and then at room temperature for 1 h. The amount of zoospores in the solution was determined by vortexing an aliquot of the zoospore solution for 30 s to induce encystment and counting the number of cysts with a hemocytometer.

### Plant Inoculation

Plants were inoculated in the soil near the base of the plant with a zoospore suspension of *P. capsici* containing 5 × 10^5^ zoospores. Plants were flooded immediately after inoculation for 24 h to allow free movement of zoospores and then maintained wet and incubated at 26°C. *P. capsici* symptoms were recorded at 10 and 14 dpi for *N. edwardsonii* and at 3, 6, and 9 dpi for *N. benthamiana* and *N. clevelandii*. For recording symptoms, the following scale of infection ranging from 0 to 3 was used: 0 = healthy, with no visible symptoms; 1 = wilting or infection covering up to 20% of the plant; 2 = infection covering up to 80% of the plant; 3 = infection covering up to 100% of the plant. Since progression of the infection produced by *P. capsici* can differ depending on the plant species infected, we used this scale of infection because of its simplicity and reproducibility for recording infection in *N. edwardsonii*.

### Virus-Induced Gene Silencing

Initial screening of VIGS-amenable species was performed using the pTV00 system as described (Ratcliff et al., 2001). Follow-up experiments using VIGS were performed as described using the pTRV2 vector (Liu et al., 2002a). Constructs pTRV2-*EDS1*, pTV00-*SGT1*, and pTRV2-*Gus* have been described before (Liu et al., 2002a; Peart et al., 2002b; Tameling and Baulcombe, 2007). To construct pTRV2-*I2*, a fragment of 840 nucleotides of the *I2* gene corresponding to the nucleotide binding site (NBS) region was amplified from *N. tabacum* DNA using primers I2-2F and I2-2R (Couch et al., 2006). A multiple alignment of 44 distinct NBS sequences derived from the tobacco *I2* multigene family (Couch et al., 2006) downloaded from the GenBank showed a high similarity (**Supplementary Figure S1**). Thus, for VIGS of *I2* the PCR products were sub-cloned into pGEM-T easy vector (Promega) and a random clone was sequenced and subsequently cloned in sense orientation into the *EcoR*I site of the pTRV2 vector (Liu et al., 2002a).

Virus-induced gene silencing assays were carried out in *N. edwardsonii, N. benthamiana*, and *N. clevelandii*. Three-week-old plants were infiltrated with the *Agrobacterium* strain GV3101 and C58C1 harboring TRV1 along with *Agrobacterium* harboring pTRV2-*EDS1*, pTRV2-*I2*, or pTV00-*SGT1*. Agroinfiltration of VIGS constructs was carried out as previously described (Collier et al., 2011). Plants were kept in the greenhouse for 3 weeks after infiltration and then moved to a restricted-access confinement facility for inoculation with *P. capsici*.

### Transgenic Plants

Transformation of *N. tabacum* with the RNAi construct NtI2hp was performed at the Plant Transformation Core Laboratory of the Boyce Thompson Institute for Plant Research, Ithaca, NY, USA. Twenty independent lines were obtained and tested for resistance to *P. capsici*. For plant transformation, the binary RNAi construct NtI2hp was generated using the RNAi gateway vector pHellsgate (Wesley et al., 2001) and introduced into *Agrobacterium* strain EHA105 by electroporation.

## Author Contributions

Conceived and designed the experiments: JV-A and PM. Performed the experiments: JV-A and JS-S. Analyzed the data: JV-A, HS-B, JS-S, and PM. Contributed reagents/materials/analysis tools: JV-A, HS-B, and PM. Wrote the paper: JV-A and PM.

## Conflict of Interest Statement

The authors declare that the research was conducted in the absence of any commercial or financial relationships that could be construed as a potential conflict of interest.

## Footnotes

1 http://genome.jgi.doe.gov/Phyca11/Phyca11.home.html

## Supplementary Material

FIGURE S1 **Alignment of the tobacco I2-NBS sequence used for VIGS in *N. edwardsonii* and for transformation of *N. tabacum* plants with the 44 sequences of the NBS region of *I2* homologs from tobacco reported previously (Couch et al., 2006).** The alignment showed an overall similarity of 78.5%. One of the more conserved blocks of the alignment (containing at least 25 identical nucleotides in all sequences) is presented to show the potential of VIGS and RNAi to silence most of the members of the *I2* family.

FIGURE S2 **VIGS of *SGT1* in *N. edwardsonii* results in lethality.** Three-week-old plants were infiltrated with *Agrobacterium* carrying TV-*SGT1* or pTV00 along with *Agrobacterium* carrying pBintra6 (RNA I), as indicated. Non-TRV-infected plants are shown for comparison. Photographs were taken 3 weeks after initiation of VIGS.

FIGURE S3 **Resistance to *P. capsici* is compromised in transgenic tobacco leaves expressing a RNAi construct to silence *I2* genes.** Infection assay with *P. capsici* in transgenic tobacco lines expressing a hairpin of I2 was carried out in detached leaves of 4-week-old plants. The graph shows the infection size (diameter) in leaves from wild-type (WT) and the different transgenic lines (L1–L20) inoculated with *P. capsici* mycelium. Data was collected at 2 dpi and the average of measurements from three to six independent experiments is shown. Error bars represent standard errors.

FIGURE S4 **Resistance to *P. capsici* is compromised in transgenic tobacco plants expressing a RNAi construct to silence *I2* genes.** Infection assay with *P. capsici* in transgenic tobacco lines expressing a hairpin of I2 was carried out in 2-week-old plants. The graph shows the ratio of infected plants/total inoculated plants. This is a representative experiment in which 18 to 24 plants were used of each of the WT and the different transgenic lines (L1–L20) and the data represents the absolute number of plants tested. Plants were inoculated in the soil near the stem with a suspension of 18 × 10^4^ zoospores of *P. capsici*. Data was collected at 6 dpi.

FIGURE S5 ***P. capsici* Avr3a-triggered HR in *N. edwardsonii* is not compromised in *EDS1*- and *I2*-silenced plants. (A)** Leaves of *N. edwardsonii* and *N. benthamiana* VIGSed for *I2* were agro-infiltrated with Avr3a1 or empty vector (pBin61). Photographs were taken at 5 dpi. **(B)** Percentages of infiltrated sites showing HR in non-VIGSed *N. edwardsonii* plants and plants VIGSed with TRV carrying inserts of GUS, *EDS1*- and *I2*. HRs were scored at 5–8 dpi and error bars indicate standard deviation from three independent experiments.
