# Supplementary figures and images for: Non-host Plant Resistance against Phytophthora capsici Is Mediated in Part by Members of the I2 R Gene Family in Nicotiana spp
Source: Front Plant Sci. 2017 Feb 15;8:205. doi: 10.3389/fpls.2017.00205 (PMC5309224; doi:10.3389/fpls.2017.00205)

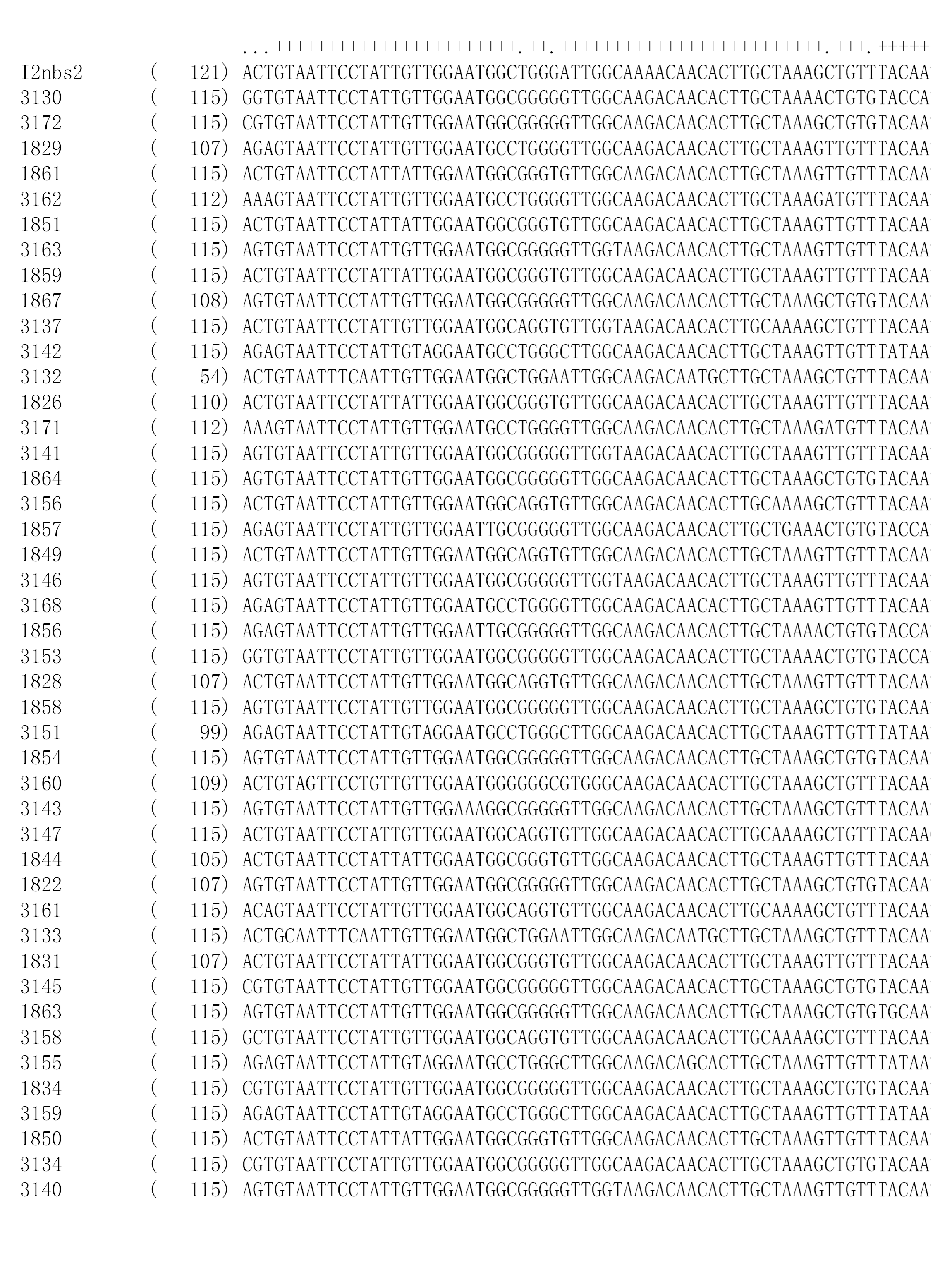

Supplement: FIGURE S1 — Alignment of the tobacco I2-NBS sequence used for VIGS in N. edwardsonii and for transformation of N. tabacum plants with the 44 sequences of the NBS region of I2 homologs from tobacco reported previously (Couch et al., 2006). The alignment showed an overall similarity of 78.5%. One of the more conserved blocks of the alignment (containing at least 25 identical nucleotides in all sequences) is presented to show the potential of VIGS and RNAi to silence most of the members of the I2 family. [file Image_1.TIF]

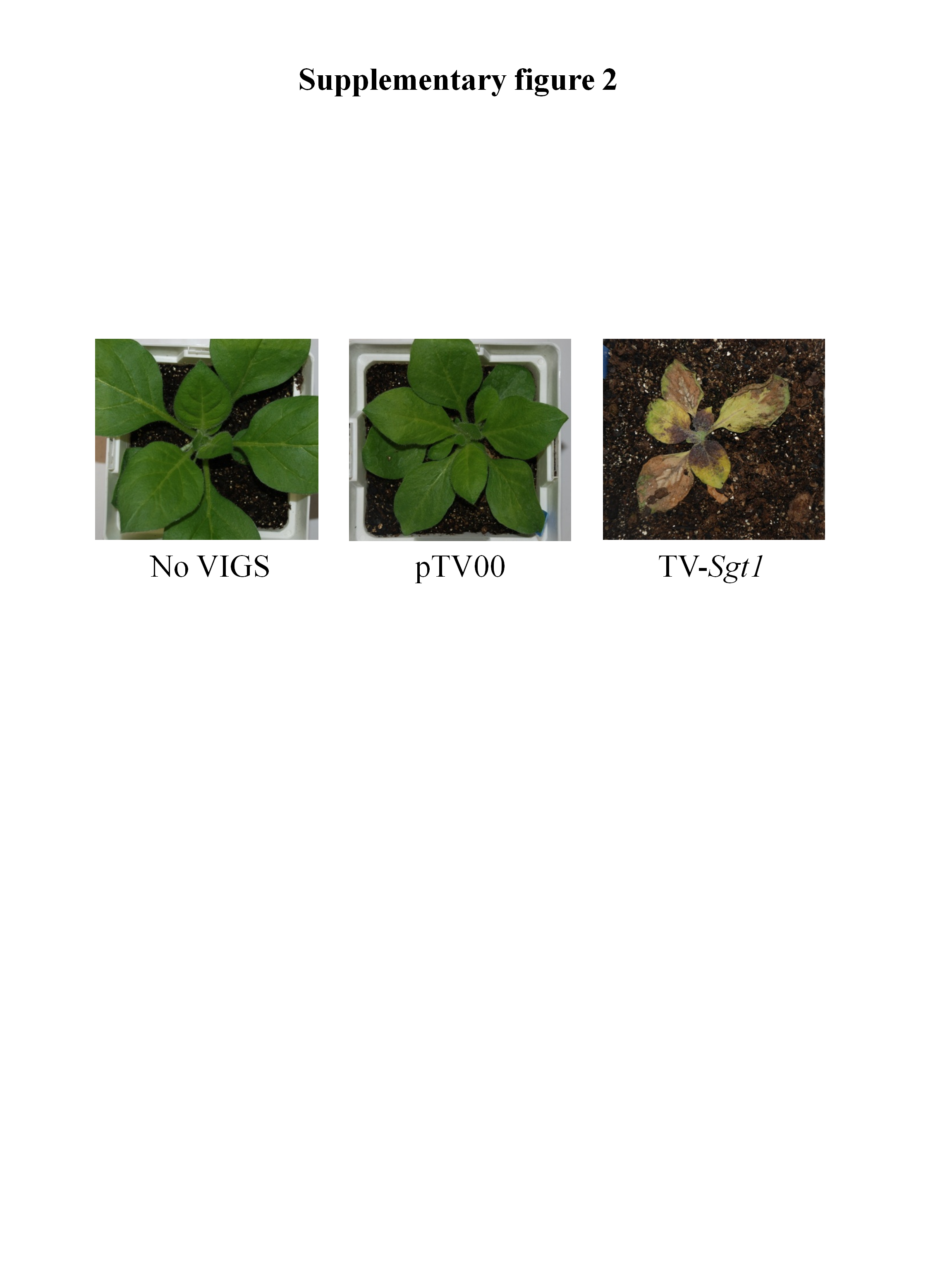

Supplement: FIGURE S2 — VIGS of SGT1 in N. edwardsonii results in lethality. Three-week-old plants were infiltrated with Agrobacterium carrying TV-SGT1 or pTV00 along with Agrobacterium carrying pBintra6 (RNA I), as indicated. Non-TRV-infected plants are shown for comparison. Photographs were taken 3 weeks after initiation of VIGS. [file Image_2.TIF]

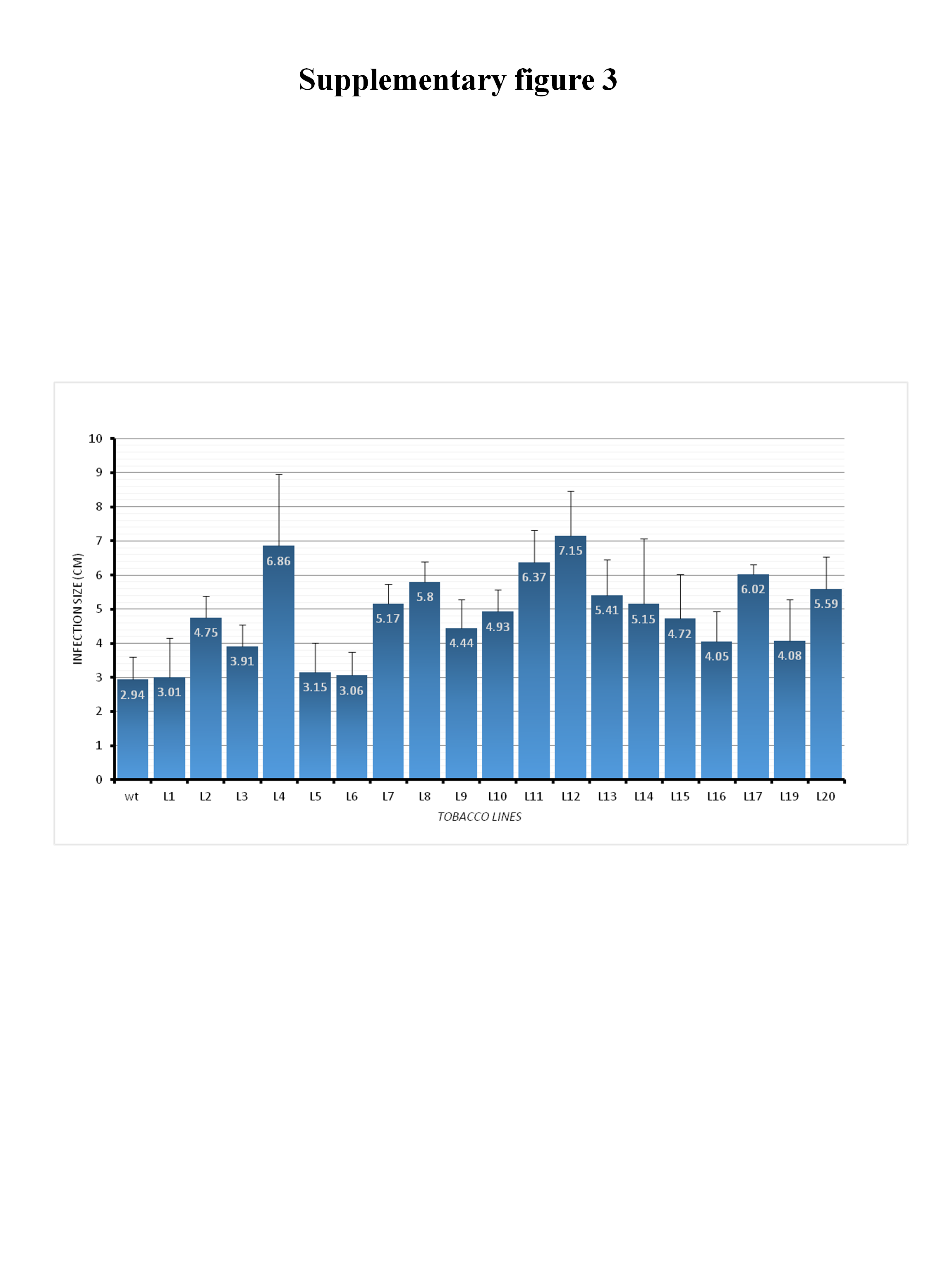

Supplement: FIGURE S3 — Resistance to P. capsici is compromised in transgenic tobacco leaves expressing a RNAi construct to silence I2 genes. Infection assay with P. capsici in transgenic tobacco lines expressing a hairpin of I2 was carried out in detached leaves of 4-week-old plants. The graph shows the infection size (diameter) in leaves from wild-type (WT) and the different transgenic lines (L1–L20) inoculated with P. capsici mycelium. Data was collected at 2 dpi and the average of measurements from three to six independent experiments is shown. Error bars represent standard errors. [file Image_3.TIF]

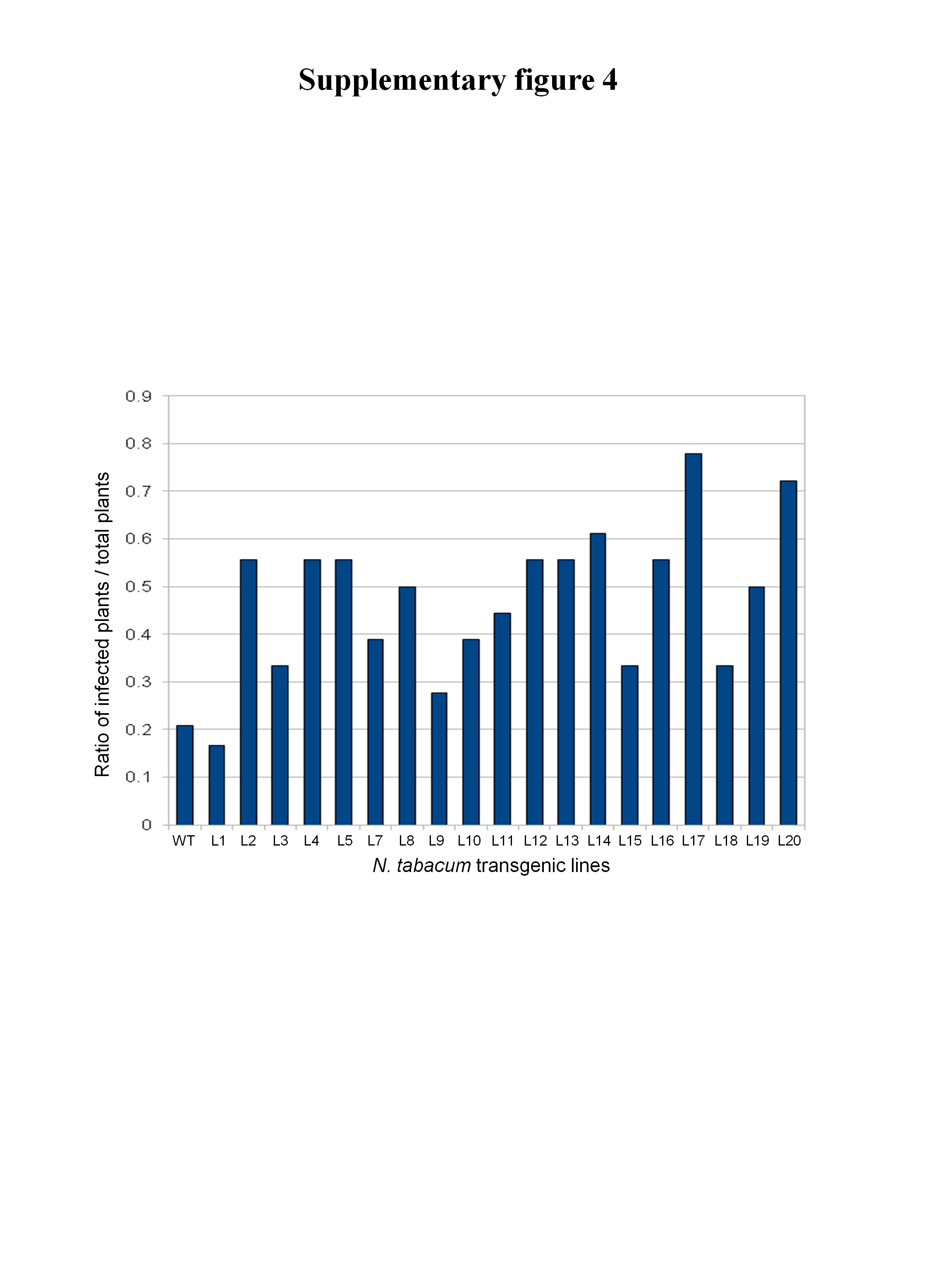

Supplement: FIGURE S4 — Resistance to P. capsici is compromised in transgenic tobacco plants expressing a RNAi construct to silence I2 genes. Infection assay with P. capsici in transgenic tobacco lines expressing a hairpin of I2 was carried out in 2-week-old plants. The graph shows the ratio of infected plants/total inoculated plants. This is a representative experiment in which 18 to 24 plants were used of each of the WT and the different transgenic lines (L1–L20) and the data represents the absolute number of plants tested. Plants were inoculated in the soil near the stem with a suspension of 18 × 104 zoospores of P. capsici. Data was collected at 6 dpi. [file Image_4.TIF]

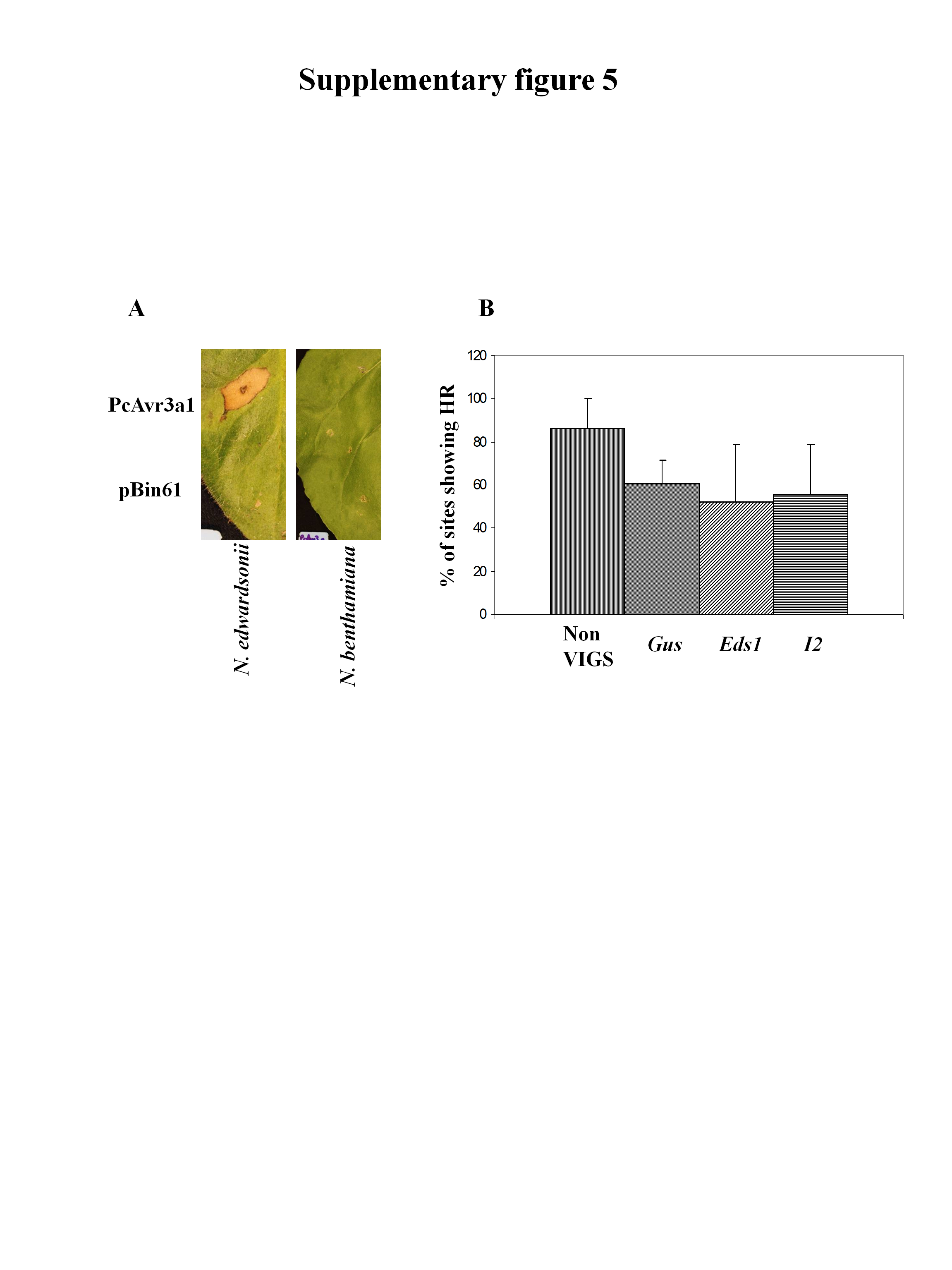

Supplement: FIGURE S5 — P. capsici Avr3a-triggered HR in N. edwardsonii is not compromised in EDS1- and I2-silenced plants. (A) Leaves of N. edwardsonii and N. benthamiana VIGSed for I2 were agro-infiltrated with Avr3a1 or empty vector (pBin61). Photographs were taken at 5 dpi. (B) Percentages of infiltrated sites showing HR in non-VIGSed N. edwardsonii plants and plants VIGSed with TRV carrying inserts of GUS, EDS1- and I2. HRs were scored at 5–8 dpi and error bars indicate standard deviation from three independent experiments. [file Image_5.TIF]
