# Supplementary material for: Non-host Plant Resistance against Phytophthora capsici Is Mediated in Part by Members of the I2 R Gene Family in Nicotiana spp
Source: Front Plant Sci. 2017 Feb 15;8:205. doi: 10.3389/fpls.2017.00205 (PMC5309224; doi:10.3389/fpls.2017.00205)
Supplement: Supplementary file 6 [file Table_1.DOCX]

**Supplementary Table 1. Amenability of *Nicotiana* species to TRV-based VIGS.**

|  | **TRV-based VIGS of *sulfur* gene** (magnesium chelatase) | **Susceptible to *P. capsici*** (Vega-Arreguin, et al. 2014) |
| --- | --- | --- |
| *N. benthamiana* | Yes^a^ | Yes |
| *N. tabacum* 'Samsun NN' | No^b^ | No |
| *N. affinis* | Yes | nd |
| *N. alata* | Yes | nd |
| *N. glutinosa* | Ni^c^ | No |
| *N. clevelandii* | Yes | Yes |
| *N. edwardsonii* | Yes | No |
| *N. rustica* | Ni | Yes |
| *N. biglolvii* | Yes | nd |
| *N. sylvestris* TW137 | Ne^d^ | nd |
| *N. sylvestris* 0067-50568 | Ne^e^ | nd |
| *N. sylvestris* TW136 | Ne | No |
| *N. macrophylla* | Ni | nd |
| *N. attenuata* | Yes | nd |
| *N. suaveolensis* | Ni | nd |
| *N. tabacum* 'Petite Havana' | Ni | nd |
| *N. tabacum* 'Virginia bright' | Ni | nd |
| *N. tabacum* 'Virginia goldleaf' | Ni | No |
| *N. langsdorfii* | Ni | nd |
| *N. otophora* | Ni | Yes |
| *N. knightiana '*899G' | Sick^f^ | nd |
| *N. undulata* | Ni | No |
| *N. longiflora* | Ne | nd |
| *N. plumbaginifolia* | Ni | No |

^a^Yes : good VIGS of sulfur gene, ^b^No : no VIGS of sulfur gene, ^c^Ni : not apparent infection by TRV, ^d^Ne : necrosis with little or no bleaching, ^e^*N. Sylvestris*: poor infection with necrotic flecks, ^f^*N. knightiana*: plants deformed by TRV.
